# Radial Recurrent Artery: Autologous Patch Graft for Acute Brachial Artery Laceration

**DOI:** 10.7759/cureus.10682

**Published:** 2020-09-27

**Authors:** Kendall J Keck, Thomas J Adams, Kristopher M Day

**Affiliations:** 1 Plastic Surgery, University of Iowa Hospitals and Clinics, Iowa City, USA; 2 Surgery, Marshall University Joan C. Edwards School of Medicine, Huntington, USA

**Keywords:** peripheral vascular surgery, upper extremity trauma, hand surgery, upper extremity reconstruction

## Abstract

Brachial artery injury is the most common vascular disruption in upper extremity penetrating trauma, usually treated by primary repair or saphenous vein interposition graft. We report the case of a young male who presented after assault with stab wound to the right antecubital fossa, an asymmetric vascular exam, and unknown depth contaminated wound that warranted operative exploration. We performed open exploration through a triangular flap extension of his oblique linear laceration for both exposure and flexor surface scar contracture prophylaxis. Exploration revealed brachial artery laceration with loss of approximately 30% of vessel circumference proximal to the radial and ulnar artery bifurcation. A near-complete transection of the recurrent radial artery was also present, leading to the decision to sacrifice this vessel for use as an autologous patch graft of the injured brachial artery. Distal vascular flow was re-established, and the vessel was slightly ectatic with no evidence of stenosis. Patient suffered no complications and was discharged at post-operative day four after perioperative heparin drip on anti-platelet therapy. Autologous patch grafting in the acute setting is a less-often considered surgical option that is effective for arterial bifurcation reconstruction, which may be employed through the sacrifice of injured and redundant local branch vessels. Patch grafts are commonly utilized in planned vascular surgery, such as carotid endarterectomy, but this is the first report of autologous patch graft to an acute brachial artery injury. By combining knowledge of the lateral arm flap with the plastic surgery principles of “like replaces like”, this technique avoids the stenosis associated with primary repair, the multiple anastomoses necessary for interposition grafting, the need for a secondary donor site, and provides a theoretical blood-flow advantage.

## Introduction

Vascular injuries to the upper extremity represent 30-50% of peripheral vascular injuries, the majority of which are penetrating trauma involving the brachial artery [1]. The incidence in the United States is difficult to characterize, but can be inferred based on studies involving a single trauma center. Franz et al., reports 30 upper extremity arterial injuries in a two-year period at a large urban level 1 trauma center [2]. Sixty percent were penetrating and 40% involved the brachial artery. In another recent review of the National Trauma Databank, brachial artery was second only to iliac artery in incidence of injury in trauma [3]. The brachial artery is especially vulnerable to damage because of its location within the antecubital fossa, which is less protected by muscle and soft tissue than the upper arm or the forearm [4].

Arterial injuries to the upper extremities are diagnosed bedside by clinical exam and doppler ultrasonography. Angiography may be performed, but this is a time-consuming procedure that may not be feasible in a situation requiring potentially limb-saving surgery. Surgical repair of these injuries is traditionally accomplished by primary repair of a laceration, resection with end-to-end anastomosis, or saphenous vein interposition grafting [5]. Bovine carotid patch and endovascular techniques have also been described but are not well studied or described in brachial artery injuries [6,7].

Patch angioplasty is scarcely described in the trauma literature, but is commonly used in elective vascular surgery procedures [8]. The classic example is carotid endarterectomy. Randomized prospective trials and meta-analyses have demonstrated improved rates of stenosis with a patch as compared to primary closure of the carotid arteriotomy [9].

We report the case of an autologous patch angioplasty utilized to repair a brachial artery laceration that occurred secondary to penetrating trauma. Based on previous literature, this approach is seldom employed. However, we believe that an autologous patch can be a useful addition to the toolbox of any plastic, vascular, or trauma surgeon who encounters upper extremity vascular injuries.

## Case presentation

The patient presented to the emergency department as a 33-year-old male after an altercation that resulted in a penetrating stab wound to the right antecubital fossa. He complained of pain and numbness of the right hand. Examination was difficult due to agitation and noncompliance, but a 2 cm oblique laceration to antecubital fossa with active bleeding was observed. He had palpable but unequal distal radial pulses in the right upper extremity compared to the left. Given the high risk of vascular injury with this mechanism in this location, and the unknown cleanliness of the assault weapon, the decision was made to take the patient to the operating room for exploration and potential limb-preserving intervention.

The decision to take this patient to the operating room proved controversial. The patient lacked the so-called “hard signs” of vascular injury consisting of absent pulses, bruit or palpable thrill, active hemorrhage, expanding hematoma, or distal ischemia. The original consulting service recommended bedside closure of the laceration with close observation and serial vascular exam. However, our index of suspicion for brachial artery injury remained high enough that we decided to take the patient for exploration.

**Figure 1:**
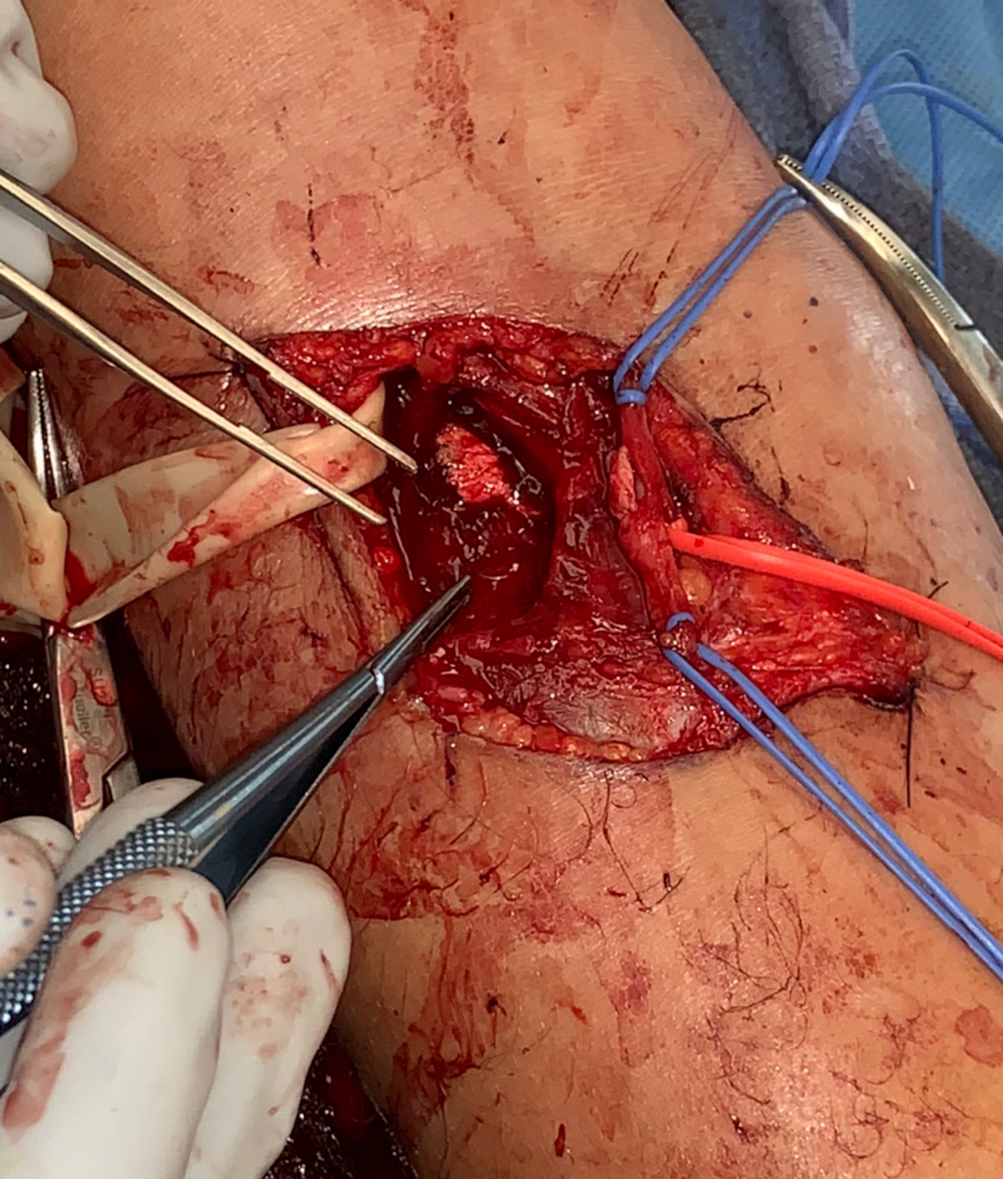
Brachial Artery Laceration Intra-operative photograph displaying the defect in the brachial artery (proximal blue vessel loop), immediately proximal to the radial (distal blue vessel loop)-ulnar (red vessel loop) bifurcation. Instruments indicate the track of the injury and site of radial recurrent artery harvest.

In the operating room the patient was found to have a weak radial pulse and absent ulnar pulse with soft compartments. A non-sterile tourniquet was inflated at the proximal upper arm to occlude blood flow during the operation. Given the oblique orientation of the laceration, the incision was extended in a z-fashion at the proximal and distal ends to prevent future flexion contracture. Careful dissection was carried down through the antebrachial fascia. Opposing triangular flaps were raised and secured using nylon stay sutures, providing adequate exposure for exploration of the antecubital fossa and evaluation of the vasculature (Figure 1).

The brachial artery suffered a linear laceration immediately proximal to the bifurcation of the radial and ulnar arteries that involved approximately 30% of the vessel circumference. This is demonstrated in Figure 1. We also observed an approximately 90% injury to the radial recurrent artery that was only attached by a small tether. Careful dissection was carried through the tunnel made by the penetrating weapon and a counter incision was made to aid in exposure, followed by copious irrigation. A series of vessel loops was placed around the brachial artery, ulnar artery, radial artery, and radial recurrent artery in order to obtain proximal and distal control. The tourniquet was released and the vessel loops were sequentially released to evaluate flow. Poor flow was noted in the brachial, radial, and ulnar arteries, but brisk flow was noted in the radial recurrent artery. The poorly flowing vessels were flushed with heparinized saline using an angiocath, and then a Fogarty catheter was inserted through the laceration to remove any clots. This was successful and adequate flow was noted through all vessels.

**Figure 2:**
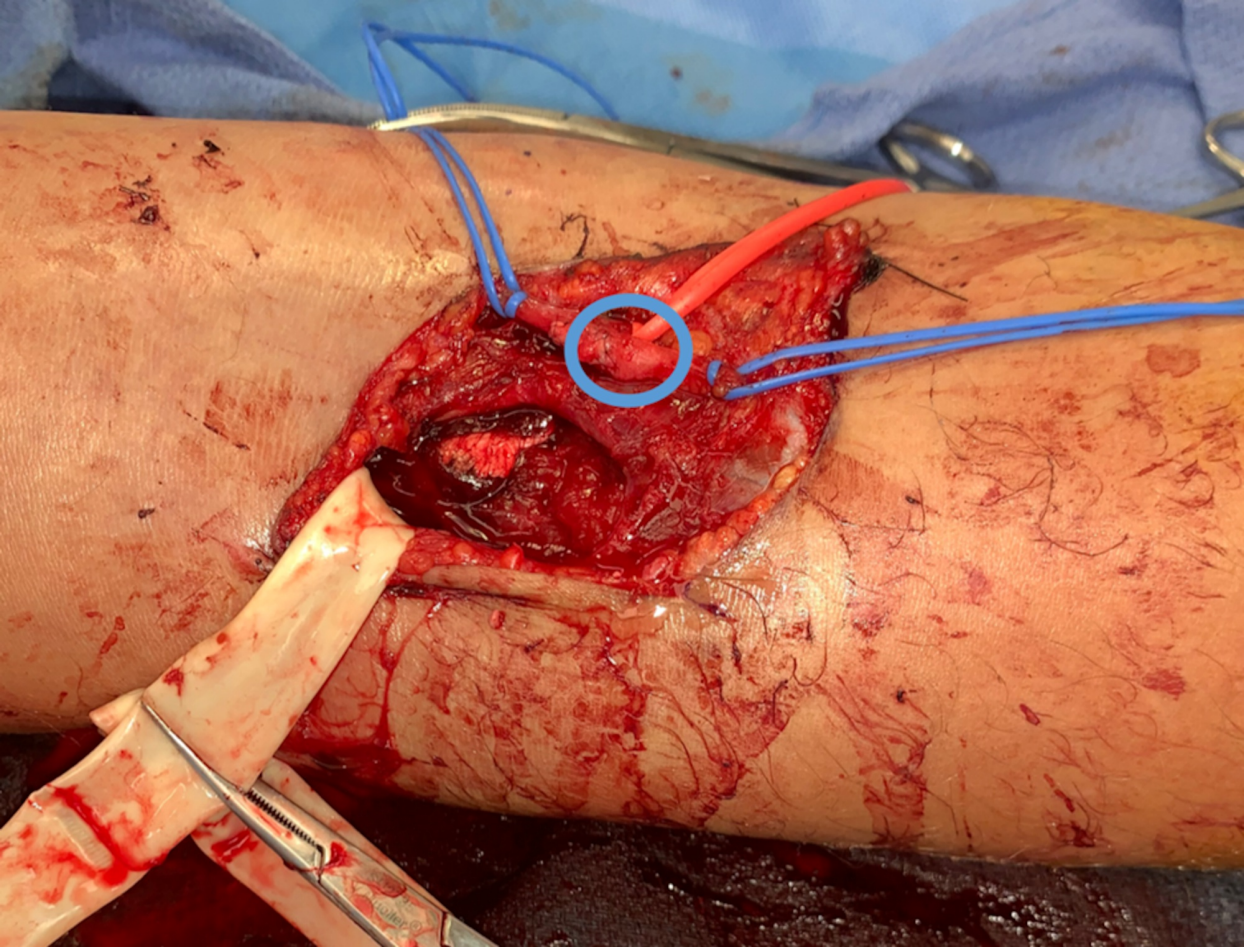
Autogenous Patch Graft Intra-operative photograph displaying the repaired brachial artery using the autologous patch graft (circled in blue) harvested from the sacrificed radial recurrent artery.

Attention was turned to repairing the brachial artery laceration. Intraoperative consultation with an upper extremity orthopedic surgeon and an experienced general surgeon was requested. As a multidisciplinary team, we decided to sacrifice and harvest the nearly transected radial recurrent artery and fashion an autologous patch graft (Figure 2) over the bifurcation of the brachial artery into the radial and ulnar arteries. The justification was that a primary closure would cause significant stenosis at a vital bifurcation and further threaten the arm. A large vessel clip was employed to ligate the radial recurrent artery 2 cm proximal to the laceration site, then a patch graft was fashioned in approximately the shape of the brachial artery defect. Proximal and distal sutures were placed using 8-0 nylon to secure in place the graft. Interrupted 8-0 nylon sutures were then placed circumferentially with maximal intimal reapproximation. This is demonstrated in Figure 2. The vessel loops were relaxed for flow to be tested through the patched vessel. One small leak was identified and repaired with another single 8-0 nylon interrupted suture. Distal flow was confirmed with intraoperative doppler (Video 1) which displayed triphasic signals in the ulnar and radial arteries at the wrist. Pulses were also palpated easily.

**Video 1:** Intraoperative Doppler Ultrasound The post-patch angioplasty doppler signal was triphasic at the end of the case over both the radial and ulnar arteries distally at the wrist.

**Figure 3:**
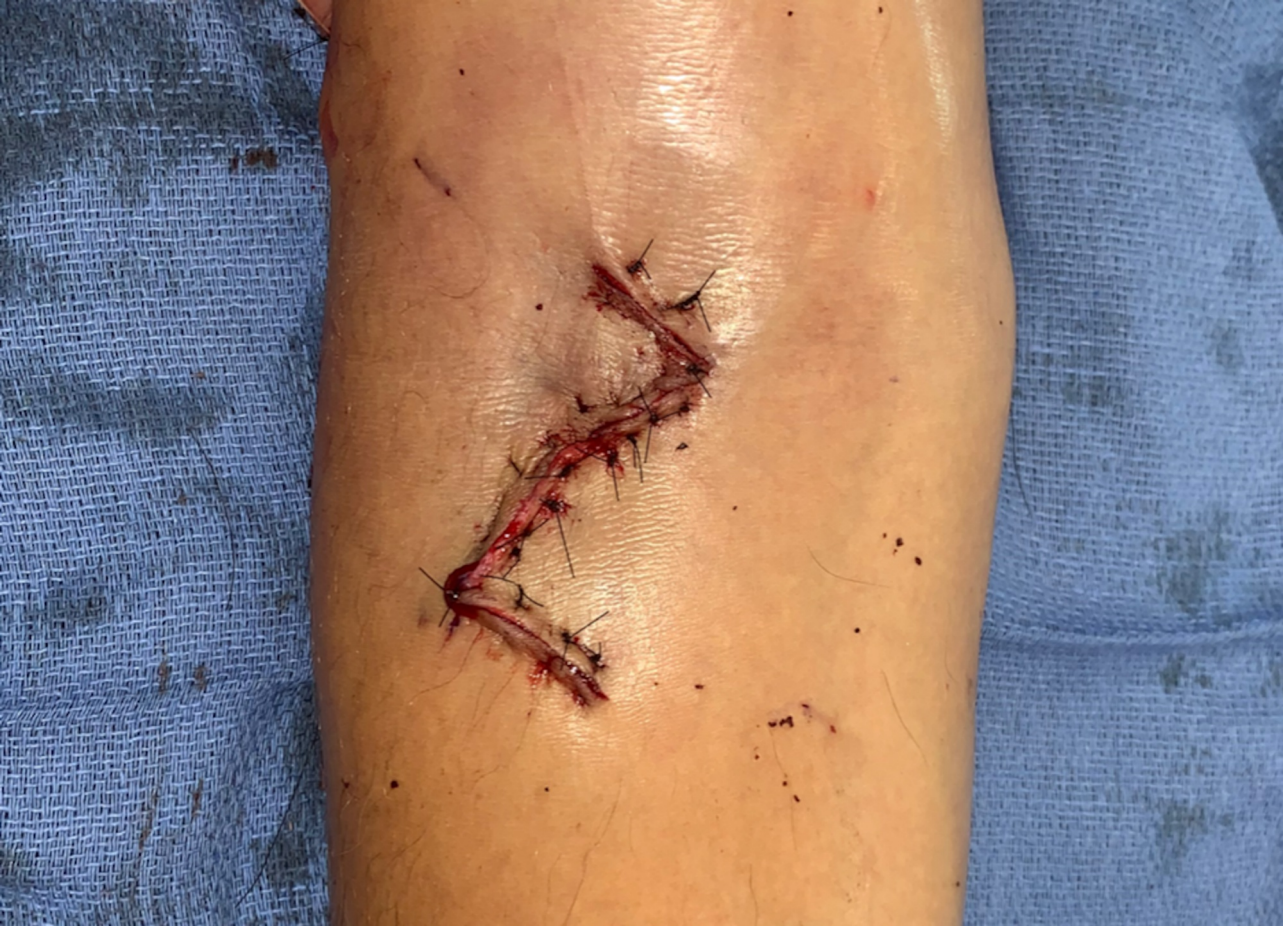
Closed Wound Intra-operative photograph displaying closed wound. Z-shaped incision was performed to provide optimal exposure and decrease the risk of flexion contracture at the antecubital fossa.

The triangular skin flaps were reapproximated with interrupted deep dermal sutures and then skin was closed with a series of horizontal and vertical mattress sutures (Figure 3). A Penrose drain was placed, and a fiberglass splint was placed to hold the patient in extension. Patient was noted to be therapeutic on heparin at the time of completion of the operation and was transferred to the post-operative care unit in stable condition.

Post-operatively, patient was transferred to the surgical ICU for serial compartment checks and neuromuscular exams which were uneventful for the duration of his stay. He was kept on a heparin drip until postoperative day three and given aspirin 325 milligrams per day. The patient left the hospital post-operative day four. He presented to the emergency department on postoperative day 11 with right arm pain that was consistent with normal postoperative course and was noted to have palpable and equal pulses in the distal right extremity with otherwise normal neurovascular exam.

## Discussion

Our suspicion for vascular injury came from the location of the laceration in the antecubital fossa, a combative patient with an unreliable clinical exam, and a subjective asymmetric pulse exam. We also felt that the patient would benefit from operative washout due to unknown cleanliness of the assault weapon. These factors lead us to perform exploratory surgery with identification of major vessel injury requiring vascular repair. This is an interesting example in which the “hard signs” of vascular injury failed to identify an injury that warranted exploration, but the “soft sign” of distal pulse asymmetry proved a sufficient indicator of vascular injury [10]. Our case was not amenable to traditional vascular repair due to the injury’s proximity to the radial and ulnar artery bifurcation. Primary repair would have likely caused significant stenosis. Interposition grafting would have required a bifurcating graft or multiple grafts, which risked too many locations for anastomotic failure. To our knowledge, this is the first reported case of utilizing patch angioplasty to treat a traumatic brachial artery injury.

On review of multiple upper extremity vascular trauma studies, ligation, primary repair, resection, and end-to-end anastomosis, or interposition grafting are all described. Pillai et al. collected data on 21 traumatic arterial injuries from 1992 to 1994 and reported 20 of those patients underwent exploration, 16 (80%) received interposition grafting, and two underwent primary repair (10%) [11]. Ergunes et al. described 58 brachial artery injuries between 1996 and 2004; 32 (55.2%) were repaired with end-to-end anastomosis, 18 (31%) with reverse saphenous vein graft, and eight (13.8%) by primary repair [12]. Ekim et al. performed surgery on 49 patients between 1999 and 2008, with 28 (57%) having an end-to-end anastomosis, interposition grafting in 15 cases (30%), and primary repair in five (10%) [5]. We elected to apply the prototypically plastic surgical principle of “like replaces like” to preserve critical vessel diameter at a bifurcation point using the novel approach of adjacent vessel salvage patch angioplasty [13].

The surgical treatment options mentioned above are generally dictated by the type of arterial injury suffered [14]. End-to-end anastomosis is usually performed in response to a complete transection of the vessel. Interposition grafting is chosen when there is significant tissue loss requiring extra material to be used to regain continuity or significant thrombosis of the vessel requiring bypass. Primary repair is used for partial lacerations. Patch angioplasty is not a replacement for complete transections or injuries requiring bypass. However, in patients with partial lacerations a patch could be a useful adjunct or replacement for the primary repair. Follow-up data concerning long-term stenosis of primarily repaired traumatic arterial injuries is simply not assessable based on our literature review. However, an abundance of data exists on the restenosis and distal thrombosis of carotid arteries following carotid endarterectomy. This data shows a clear superiority in patch angioplasty compared with primary repair [9].

Numerous materials are used for arterial repair in vascular surgery, including prosthetic grafts (Polytetrafluoroethylene [PTFE]/Dacron), autologous vein patches, and xenografts, such a bovine pericardium/peritoneum [15]. One unique aspect about our case was available patch material within the surgical field in the nearly transected radial recurrent artery. Native arteries are not typically used as material for patch angioplasty due to prohibitive donor site morbidity as compared to veins or allografts, but this was not a concern in the traumatic setting. Additionally, we knew that the sacrifice of the radial recurrent artery would be compensated for by the radial collateral circulation. The presence of a nearby native arterial donor vessel that would require ligation regardless thereby provided an opportunity to execute immediate reconstruction with optimal vascularity with the expectation of long-term patency.

## Conclusions

We describe a unique case of radial recurrent artery salvage patch angioplasty repair of a lacerated brachial artery secondary to penetrating trauma. This approach is not previously described in the trauma literature and employs concepts utilized in both vascular and plastic surgery. Further research is suggested to validate this approach, but we believe that patch angioplasty should be added to the arsenal of penetrating vascular injury repair techniques, especially near points of arterial bifurcation.
